# Routine Photography of Injuries: A Comparison Between Smartphone Cameras and Digital Single-Lens Camera—A Pilot Study

**DOI:** 10.1097/PAF.0000000000000825

**Published:** 2023-03-31

**Authors:** Arianna Giorgetti, Jennifer Paola Pascali, Guido Pelletti, Annamaria Silvestri, Elena Giovannini, Susi Pelotti, Paolo Fais

**Affiliations:** From the ∗ Unit of Legal Medicine, Department of Medical and Surgical Science, University of Bologna, Bologna; † Department of Cardiologic, Thoracic and Vascular Sciences, University of Padova, Padova, Italy.

**Keywords:** photo documentation of injuries, autopsy service, smartphone cameras, digital single lens camera

## Abstract

Ten lesions were photographed with an entry-level (HUAWEI P smart 2019), a midrange (Samsung Galaxy S8) and a high range (Apple Iphone XR) smartphone camera and with a digital single-lens camera (DSLC). Images were independently rated by 3 pathologists, based on comparison to the real lesion and “visual impact.” Difference of perceptual lightness coordinates between smartphones and the criterion standard (DSLC) was calculated.

The highest ranking for adherence to reality was obtained with DSLC, while the highest ranking for visual impact was obtained with the Iphone. The color representation better reflecting the criterion standard (DSLC) was obtained for the entry-level smartphone.

All the devices allow to assess the general features (ie, the color, the shape, and the main characteristics) of an injury during a forensic autopsy. However, results might be different when photos are obtained in suboptimal, such as low-light, conditions. Moreover, images acquired through a smartphone camera might be unsuitable for later image exploitation, such as enlargement of a portion of the image to magnification of a detail, which seemed not relevant when the photo was shot. Only a raw image, acquired using a dedicated camera and deactivating images manipulation software, might allow the preservation of the true data.

The utilization of photography is a milestone in autopsy service, for the illustrative documentation of postmortem examinations, to preserve the evidentiary value of injuries for the future and to create demonstrative images for courtroom presentation.^1,2^ During forensic examinations, the ones who take pictures are usually pathology residents, who are also responsible for the photo documentation.^3^ In the past, when film photography was the only method of documentation, the pathologist could not see the result until after postprocessing, which could take days. Accordingly, the number of pictures taken at autopsy was limited and photographs were supportive, but not of primary importance.^1^ With the emergence of digital photography, digital cameras have been increasingly used during forensic procedures, with an increase in documented examinations.^3^ The most important innovation that digital photography has provided in terms of usefulness is the ability to immediately view the results of taking a picture, together with a rapid take of multiple images.^3^ These advantages have turned digital photography a primary method of visual documentation for autopsy practitioners.^1^

In the last decade, the persistent improvement of technologic features of mobile phones, including upgrades of smartphone cameras, allowed the fast and easy capture of high-resolution images. However, smartphone cameras are tiny if compared with a real digital camera and this physical limitation is relevant for image quality. Actually, smaller sensors capture smaller amounts of light and the amount of light influences the image quality.^4^ However, that is where computational photography comes in. By combining machine learning, computer vision, and computer graphics with traditional optical processes, computational photography aims to enhance what is achievable with traditional digital cameras. For example, one of the most powerful approaches by software is to capture multiple images, even using multiple in-built cameras, in a short interval and merge them to synthesize into a result image. This process is usually automatized and almost unnoticed from the user. Moreover, different dedicated algorithms allow to enhance the features of the resulting image that is immediately available: multiframe noise reduction allow to reduce noise, high dynamic range (HDR) compression allow to optimize exposure, and superresolution allow to increase resolution.^5^ In modern smartphones, enhancement of snapshots may be achieved also through images analysis techniques, powered by artificial intelligence and deep neural networks. Image classification is a technology that aims to estimate the category to which the image belongs allowing to change shooting settings based on the result of classification. In particular, advances in image analysis have been achieved for facial filtering. Face landmark detection enables the localization of different regions of a face, including skin, facial hair, eyes, lips, and enabling different filtering parameters. For example, a selfie can be enhanced by smoothing the skin to diffuse wrinkles, freckles, etc, while still keeping the eyes, hair, and facial hair sharp.^5^

Modern digital methods, including these functions to ameliorate pictures, have grown complexity in the scenario of autopsy photography, enabling a consumer level postprocessing, but sometimes might lead to lower image interpretability and reliability.^1^ To provide a “good” picture, which might be useful in courtroom, on the other hand processing, such as basic darkroom techniques necessary for proper image formation, is usually almost essential and thus should not be considered per se a bad thing.^1^

The aim of this article is to test and compare photos of injuries acquired with entry-level, midrange, and high-range smartphone cameras, with images acquired through a digital single-lens camera (DSLC) and with the macroscopic inspection of the real lesion to assess the adherence to reality and visual impact of the photographed injuries. Comparison of digital color representation between smartphone cameras and DSLC was also performed.

## MATERIALS AND METHODS

### Study Design

The study was performed on 10 skin and soft tissues lesions selected during 5 forensic autopsies. For each lesion, the study included the following: a photographic session, in which 4 different photographic devices were used for injury documentation by a 15-year experienced forensic pathologist; 2 rating sessions, in which 3 forensic pathologists rated the images acquired with each device on the basis of 2 parameters: images' adherence to reality and visual impact. Lastly, images were compared in terms of digital color representation.

### Photographic Session

Skin and soft tissues injuries were photographed by a single operator with the following devices: entry-level smartphone camera (HUAWEI P smart 2019) (device A); midrange smartphone camera (Samsung Galaxy S8) (device B); high range smartphone camera (Apple Iphone XR) (device C) and DSLC (Nikon D3550 equipped with AF-P Nikkor 18–55 mm 1:3.5–5.6 G) (device D).

All pictures were shot under optimal light conditions with a colorimetric scale as a reference for comparison.^6^ The physician was asked to follow the standardized routine for postmortem forensic documentation, that is, skin surface was cleaned before documentation and the photograph was taken perpendicular to the injury. The camera app default settings were not modified and snapshots were performed in “auto-mode.” The DSLC was held by hands by the operator, with automatic mode, and the autofocus was used. The picture was obtained by visualizing the lesion through the viewfinder, and the preview of the image was immediately observed on the built-in LCD screen. General information about images are available in Table 1.

**TABLE 1:** Information About Images Acquired Through Each Device

	Entry-Level Smartphone	Midrange Smartphone	High-Range Smartphone	Digital Single Lens
Dimension, px	3120 × 4160	1960 × 4032	3024 × 4032	6000 × 4000
Resolution, dpi	72	72	72	NA
File format	Jpeg	Jpeg	Heic	NEF
Color space	RGB	RGB	RGB	RGB
Color profile	sRGB IEC61966-2.1	sRGB IEC61966-2.1	Display P3	Display P3
White balance	Auto	Auto	Auto	Auto
HDR	Auto	Auto	Auto	Auto
Exposure	Auto	Auto	Auto	Auto
Flash	Auto	Auto	Auto	Auto

### Ranking Sessions

#### Ranking Session I

Right after each external examination, 3 forensic pathologists were asked to independently and blindly rate the photographs. Ranking took place in the autopsy room, after images were downloaded from the smartphones and from the DSLC memory card into a single Apple MacBook equipped with an LCD monitor (RGB color space and 1440 × 900 resolution), to limit issues related to screen calibration settings. Images were visualized by the macOS “Preview” default app with fixed display settings (maximum brightness) under the same lighting conditions.

Manipulation of images was avoided in this phase except for the removal of any information, such as watermarks or identifiable filenames, which would otherwise allow to understand which device was used for capturing each image, aimed to guarantee blind ranking. Images of each lesion were thus randomly submitted to each rater and were directly compared with the true lesion on the corpse. A score from 1 to 4 was attributed to each image, being “1” the most “adherent to reality” picture and “4” the least “adherent to reality.”

#### Ranking Session II

After a week, the same raters re-evaluated the images of the 10 lesions, blindly and independently rating for the visual impact, from 1 or “best visual impact” (the most pleasing photo) to 4 “lowest visual impact” (the most displeasing photo).

### Comparison of Digital Color Representation

Aiming to objectively measure differences between images photographed with each different device, comparisons were also performed using the Commission Internationale de l'Éclairage (CIE) color space. To represent the perceived magnitude of color differences and to calculate Euclidean distances in the color space, CIE has defined some coordinates: perceptual lightness (L) and “a” and “b,” creating the so-called Lab color space.^7^ In the present study, formerly, the images of each lesion selected for the previous ranking sessions phase were copied and pasted as layers in a single GIMP XCF image at 300 ppi resolution. Then, each layer was scaled down until at superimposition, the dimensions of the areas of interest (ie, the lesion) were equal. Thus, L-a-b coordinates defined by CIE were measured on each image using a specific software (the Open Source image editor GIMP) and focusing on specific areas of interest, namely, the lesion. Areas of interest were analyzed through the GIMP eyedropper tool sampling the average values of a 101 × 101-pixel selected area (see Supplemental Digital Content 1, http://links.lww.com/FMP/A48).

### Statistical Analysis

For the parameters of “adherence to reality” and “visual impact,” mean and median ranking values obtained for lesions 1 to 10 across the 3 raters were calculated, as well as the number of votes for each scoring.

The agreement among the raters, for each device, was calculated on the basis of the Krippendorff α reliability, which is useful for interval, multicoders, and small sample size data.^8^ A Krippendorff α reliability value ≥ 0.667 was considered as a good interrater agreement.^8^

Finally, for each lesion, L-a-b coordinates obtained with each device were compared with those obtained with the DSLC (device D), considered as the criterion standard. This allows, by using a predefined tool,^9^ to calculate a ΔEab, which is used in graphics and colorimetry to evaluate the perceptual differences between 2 colors.^8^ Mean and median of ΔEab were calculated for each device. ΔEab was considered acceptable when less than 5.

For all statistical analyses, a *P* < 0.05 was set for a statistically significant result. Statistics was performed with Stata MP (version 14.0, StataCorp) and SPSS.

Figures and graphics were obtained by Adobe Photoshop (version 21.2.0, Adobe) and Prism (version 9.3.0, GraphPad Software, LLC).

## RESULTS

A total of 168 pictures were taken and revised, of which 41 taken with device A, 42 taken with devices B and C and 43 taken with device D, approximately, corresponding to 4 images for each injury and each device type.

The description of the lesions was consistent between the raters both considering the real lesion on the corpse and the pictures obtained with each different device. In summary, lesions displayed the following features.

Injury 1 consisted of a right oval orbital hematoma violet (between bluish and purple of the colorimetric scale) in color, with sharp edges, which was seen in the context of a yellowish discoloration of the face.

Injury 2 was a dry, round reddish-brown (dark red of the colorimetric scale) skin abrasion on the extensor side of the right knee.

Injury 3 was a dry patterned X-formed complex of abrasions in the thorax, of red-orange color (light brown of the colorimetric scale).

Injury 4 was a violet (between dark red and bluish of the colorimetric scale) round hematoma, localized in the left antecubital fossa, surrounded by a pink larger hematoma and centered by a millimetrical sign of acupuncture.

Injury 5 consisted of 2 round violet (bluish of the colorimetric scale) hematomas, located at the right antecubital fossa.

Injury 6 was an oval violet (between dark red and bluish of the colorimetric scale) hematoma on the left lumbar area.

Injury 7 was a violet (between dark red and bluish of the colorimetric scale) hematoma, oval in shape on the left dorsal back area.

Injury 8 corresponded to an almond-shaped incised stab wound with neat edges and bulging of the subcutaneous tissues, surrounded, at the inferior margin, by a reddish (dark red of the colorimetric scale) dry oval abrasion.

Injury 9 consisted of 2 incised wounds on the anterior surface of the knee, the first of which was and surrounded by a violet (bluish of the colorimetric scale) hematoma.

Injury 10 included several injuries of the right parietal area of the skull and, particularly, an oval ecchymotic-excoriated lesion, reddish (dark red of the colorimetric scale) in color.

**FIGURE 1:**
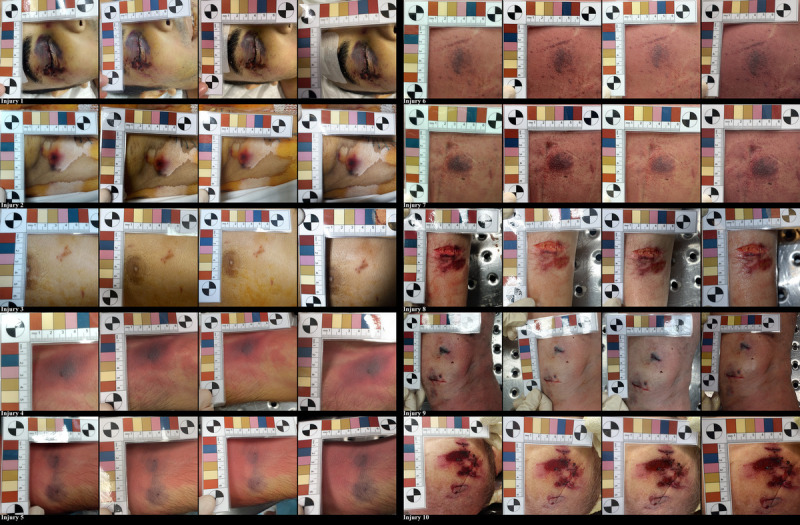
Comparison of photographs taken, from injuries 1–10 with the 4 different digital devices (from left to right): entry-level smartphone camera, midrange smartphone camera, high-range smartphone camera, and DSLC. Figure 1 can be viewed online in color at www.amjforensicmedicine.com.

A comparison of photographs taken with devices A–D is shown in Figure 1.

**FIGURE 2:**
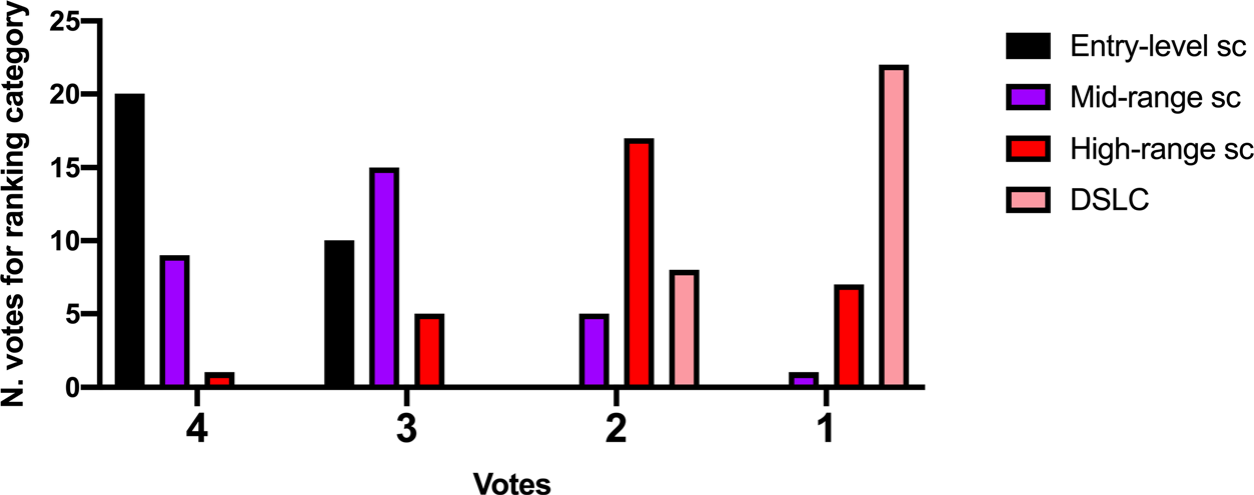
Number of votes for each device and each ranking category when rating for “adherence to reality.” Sc, smartphone camera. Figure 2 can be viewed online in color at www.amjforensicmedicine.com.

Based on the rating of the “adherence to reality,” we obtained the Table 2 showing the rating of the pictures from 1 to 4. The higher ranking, considering the mean and the median among the raters, was obtained for the DSLC, which was voted as the best one 22 times. Iphone ranked “1” only 7 times and was mostly voted as pertaining to rank “2.” Finally, the mid- and entry-level smartphone ranked respectively at the third and fourth place. More details about the scores of each single device are detailed in Figure 2.

**TABLE 2:** Results of the Ranking Performed on the Basis of the Adherence to the Reality Showing Mean and Median (Across Brackets) Ranking Obtained by Each Rater

Rater	Entry-Level Smartphone, Mean (Median)	Mid-Range Smartphone, Mean (Median)	High Range Smartphone, Mean (Median)	Digital Single lens, Mean (Median)
Rater 1	3.7 (4)	3 (3)	2 (2)	1.3 (1)
Rater 2	3.6 (4)	3.2 (3)	2 (2)	1.2 (1)
Rater 3	3.7 (4)	3 (3)	2 (2)	1.3 (1)

**FIGURE 3:**
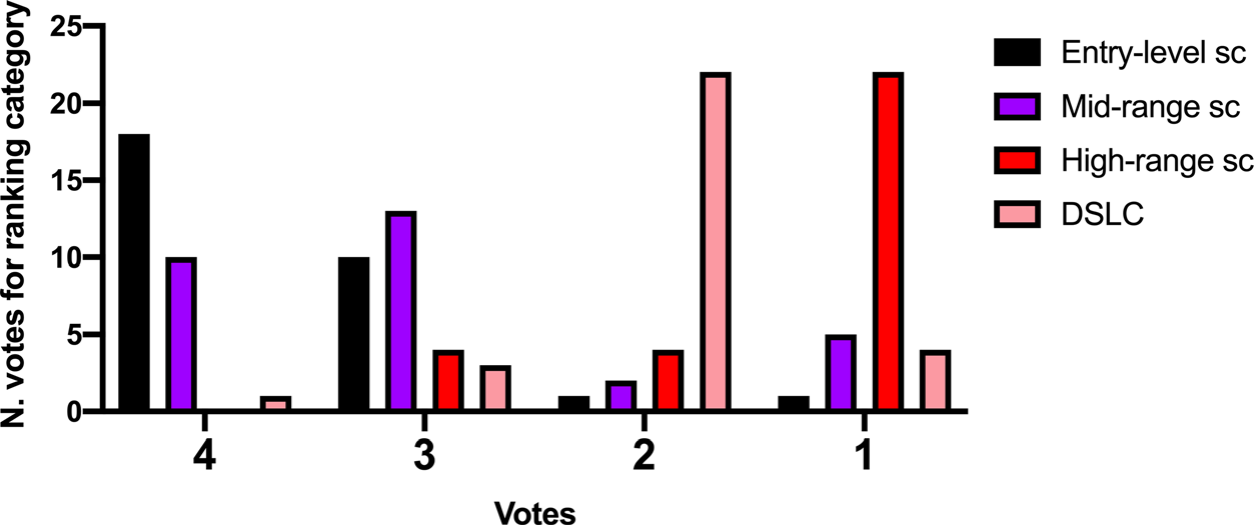
Number of votes for each device and each ranking category when rating for “visual impact.” Sc, smartphone camera. Figure 3 can be viewed online in color at www.amjforensicmedicine.com.

When re-evaluating the devices on the basis of the visual impact, the highest ranking was shown for Iphone, followed by the DSLC. Results are shown in Table 3 and Figure 3.

**TABLE 3:** Results of the Mean and Median Ranking Performed by the 3 Raters on the Basis of the Visual Impact

	Entry-Level Smartphone	Midrange Smartphone	High-Range Smartphone	Digital Single lens
Dimension, px	3120 × 4160	1960 × 4032	3024 × 4032	6000 × 4000
Resolution, dpi	72	72	72	NA
File format	Jpeg	Jpeg	Heic	NEF
Color space	RGB	RGB	RGB	RGB
Color profile	sRGB IEC61966-2.1	sRGB IEC61966-2.1	Display P3	Display P3
White balance	Auto	Auto	Auto	Auto
HDR	Auto	Auto	Auto	Auto
Exposure	Auto	Auto	Auto	Auto
Flash	Auto	Auto	Auto	Auto

A good interrater agreement was shown for the “adherence to reality” ranking, with 0.561 Krippendorff α reliability was 0.565 for entry level smartphone, 0.838 for midrange smartphone, 0.879 for high-range smartphone, and 0.670 for digital single lens. Considering all devices, a mean of 0.738 was calculated, which is consistent with a sufficient reliability.

When considering ranking session II, that is, “visual impact,” Krippendorff α reliability was 0.376 for entry-level smartphone, 0.666 for midrange smartphone, 0.555 for high-range smartphone, and 0.295 for digital single lens, with a mean of 0.473 considering all devices.

The comparison of digital color representation allowed to assess a median ΔEab of 18.0, 24.4, and 22.7 respectively, for entry-level smartphone camera, midrange smartphone camera, and high-range smartphone camera, as compared with the DSLC, which was considered the criterion standard.

## DISCUSSION

Photo documentation of postmortem examination has become sometimes equally or even more important than the report.^1^ It is especially useful to assess some features of an injury, which are difficult or impossible to explain in its entirety, including the following: its color shades, its shape, its position, and location and its relation with respect to other objects. Ideally, the photo documentation issue should be demanded to a forensic photographer who is skilled in the art of producing only the most exact, detailed photographs that record the physical evidence as objectively and accurately as possible.^10,11^ However, a forensic photographer might not always available in the forensic practice and the forensic pathologist is often claimed to provide pictures during autopsies and/or crime scene investigations sometimes using the devices available on-site, including compact cameras and even smartphones. Every photograph taken for forensic purposes can be called into court as physical evidence; hence, it is mandatory to have adequate knowledge of mechanics and technical skills for proper documentation of evidence.^12^

In our evaluation, formerly, we focused on understanding which device could provide the most adhering to reality picture. This approach could be compared with the NIIRS methodology, developed in the ‘90s to define the level of image interpretability.^13^ In particular, ranking of images in our study was limited to the first step of the NIIRS methodology, namely, the “Subjective Quality Scale.” More detailed studies taking advantage of the complete NIIRS methodology are mandatory in forensic pathology. As expected, considering the intrinsic limits of a small smartphone camera, the images that were reported most similar to real lesions through direct visual comparison during the autopsy were captured with the DSLC, followed by the high-range, midrange, and entry-level smartphone. Indeed, the DSLC is a device specifically dedicated to photography that provides larger resolution, improved camera sensor, and range of quality lenses,^10^ as well as of course a better image quality, most reflecting the human eye's perception. On the other hand, the best image at visual impact might not be necessarily the most adhering to reality. In fact, the enhancement of image features, including brightness, colors and contrast, achieved through digital image processing might improve photos making them even more pleasing than the original. This is not surprising, because the film photography era oversaturated images, particularly of people and landscapes, have been considered more aesthetically pleasing. For example, Kodachrome was considered superior to Ektachrome because of the oversaturation of skin tones (yellow/red).^14^ Likewise, for example, HDR on modern smartphones improves saturation of images, which result usually more pleasant. Indeed, in our study, the images acquired through the high-range smartphone obtained the best ratings at visual impact, overcoming the DSLC camera. Thus, when the real lesion on the corpse is no more available for direct comparison, an observer might be misled, judging an image obtained through a smartphone as the more trustworthy.

Considering digital color representation, ΔEab exceeded the limit of 5 (which is in graphics the threshold for a perceivable different color) for each tested device compared with DSLC. Surprisingly, the lowest ΔEab, better reflecting the criterion standard (the DSLC image), was obtained for the entry-level smartphone, while it was higher for midrange and high-range smartphones, despite a supposed better dotation of photographic hardware (such as more expensive camera lens and sensor). This might be related to a more performing digital image processing software leading to a better result at visual impact, however deviating from real features as perceived from the human eye.

As a matter of fact, considering overall the results of our study from a qualitative point of view, all of the devices sufficiently yield reality, when considering the basic needs of a routine autopsy documentation, and thus could be used for the task of image acquisition during autopsy. In every picture, the pathologists were able to roughly appreciate the color, the shape, and the main characteristics of the injury and, disregarding from the device, to assign the main color of the injury to the same reference color of the colorimetric scale. Nevertheless, this kind of photographic documentation might not be sufficient for later image exploitation, for example, for enlarging the image and extract some close-up of a certain detail, which might require greater resolution and dynamic range. Indeed, the need to revise and enhance a photo to obtain more information, such as further details of a lesion, is not so infrequent in routine forensic pathology. This issue could be flawed especially when resolution is low and/or extensive enhancement through software occurred. Computational photography often leads to a loss of detail in favor of a resulting more pleasant image. On the other hand, especially when more complex devices (such as DSLR dedicated cameras) are used from poorly trained residents, in addition to higher costs, complexity may be another disadvantage. The more configurable parameters there are, the more mistakes that can be made.^1,11^ Indeed, to obtain an excellent (or almost good) raw image in manual mode, using a DSLR dedicated camera is often not enough. The photographer must be aware about management of settings including at least the following: lens aperture, shutter speed, exposure, lighting, and flash.^11^ Moreover, when a not trained photographer tries a shot in manual mode using a device with poor hardware performance, such as a cheap camera or even a smartphone, results might lead to a poor result.

Even if a trained photographer is available, during a forensic autopsy, sometimes a hundred (or more) of documentary images are photographed. This could be really time consuming, requesting more than 3 hours: actually, each photograph aiming to be excellent may request as long as 120 seconds.^1^ On these grounds, sometimes cheap point and shoot cameras, rapid, cost-effective, and with quality features similar to a smartphone, can be used routinely, reserving more complex and expensive DSLRs for special shots.

Guidelines reporting quality and accreditation standards for forensic digital imaging have been developed for several purposes, especially dealing criminalistics.^15^ In this setting, the suitability of using smartphone photography have been tested sometimes leading to promising results.^16^

Most medical examiners in the United States have standard operating procedures for autopsy photography, covering photographs for identification and for injuries documentation purposes.^1^ Best practices are provided by the Photography in Custody Working Group for photographing patients in cases of sexual assaults^17^ and by the Institute of Medical Illustrators for nonaccidental injuries in children.^18^ Moreover, a guide for postmortem examination photography has been published by the Organization of Scientific Area Committees for Forensic Science.^19^

Nevertheless, imaging guidelines in forensic pathology are still lacking, and only fragmentary scientific articles or documents make some general recommendations about minimal standards recommended for a proper photographic documentation. Actually, the use of modern smartphone cameras, though sometimes discouraged, seems not banned in forensic pathology. Indeed, the use of smartphone cameras is related to undeniable advantages, such as to get immediately an image with optimized features, the chance to preview the photo on a larger screen, and even to send images to an expert for an instant telemedicine consult aimed to focus further studies on the corpse. Ethical related issues also deserve great attention. Although, in this study photos, were acquired through dedicated smartphones, personal electronic devices might be used for forensic purposes. Photos from personal devices should have strict electronic security and should be deleted as soon as possible. Moreover, modern devices are provided with cloud storage features, which are usually activated by default and should be turned off.^20^

### Limits of the Study

This study has several limitations. Formerly, although the color, the shape, and the main characteristics of each injury were fairly appreciable under our experimental condition, results might be different when photos are obtained in suboptimal, such as low-light conditions, when the software to enhance images is supposed to operate more invasively.

Moreover, the purpose of this pilot study was to evaluate the usefulness of smartphones for the documentation of general features of an injury during a forensic autopsy. Further studies are necessary to understand whether images acquired through a smartphone camera might be suitable also for later image exploitation, such as enlargement of a portion of the image to magnification of a detail, which seemed not relevant when the photo was shot.

In addition to technical issues during the shooting phase, human factors may also affect the evaluation of an image. Considering this experimental setting, biases related to perceptual constancy should not be overlooked. Perceptual constancy is the perception of an object or quality as constant even though our sensation of the object changes.^21^ There are several types of visual perceptual constancies, including the following: size, shape, color, lightness, location, and distance constancy. In this study, the ranking phase was performed comparing different images for each lesion (and to the real lesion) in a narrow timeframe. During this task, the comparison of main characteristics might be influenced from visual perceptual constancies, particularly considering color, and lightness of each image.^22^

## CONCLUSIONS

Today, the use of smartphones and smartphone cameras displays many undoubtful advantages over DSLC. Nevertheless, the use of these devices may lead to lower image interpretability and reliability especially when snapshots are performed under suboptimal conditions and the in-built digital imaging enhancing software operate more invasively to overcome intrinsic hardware limitation related to the miniaturization of camera optics. Moreover, devices that display sufficient quality for routine and general photo documentation of autopsies might not allow later image exploitation and detailed analyses. These issues in image documentation should not be overlooked, especially when digital photography is presented to the court. Based on the results of our study, we suggest a prudent approach to the use of a smartphone for digital photography during a forensic autopsy, also depending on the purpose of its documentation. When the subject of a photography is supposed to be essential for investigations, we suggest to complement photo documentation through a dedicated camera. Indeed, only a raw image, acquired using a dedicated camera and deactivating images manipulation software, might allow the preservation of the true data.

## Supplementary Material
