# Supplementary material for: Routine Photography of Injuries: A Comparison Between Smartphone Cameras and Digital Single-Lens Camera—A Pilot Study
Source: Am J Forensic Med Pathol. 2023 Mar 31;44(2):83–9. doi: 10.1097/PAF.0000000000000825 (PMC10184793; doi:10.1097/PAF.0000000000000825)
Supplement: Supplementary file 1 [file fmp-44-83-s001.docx]

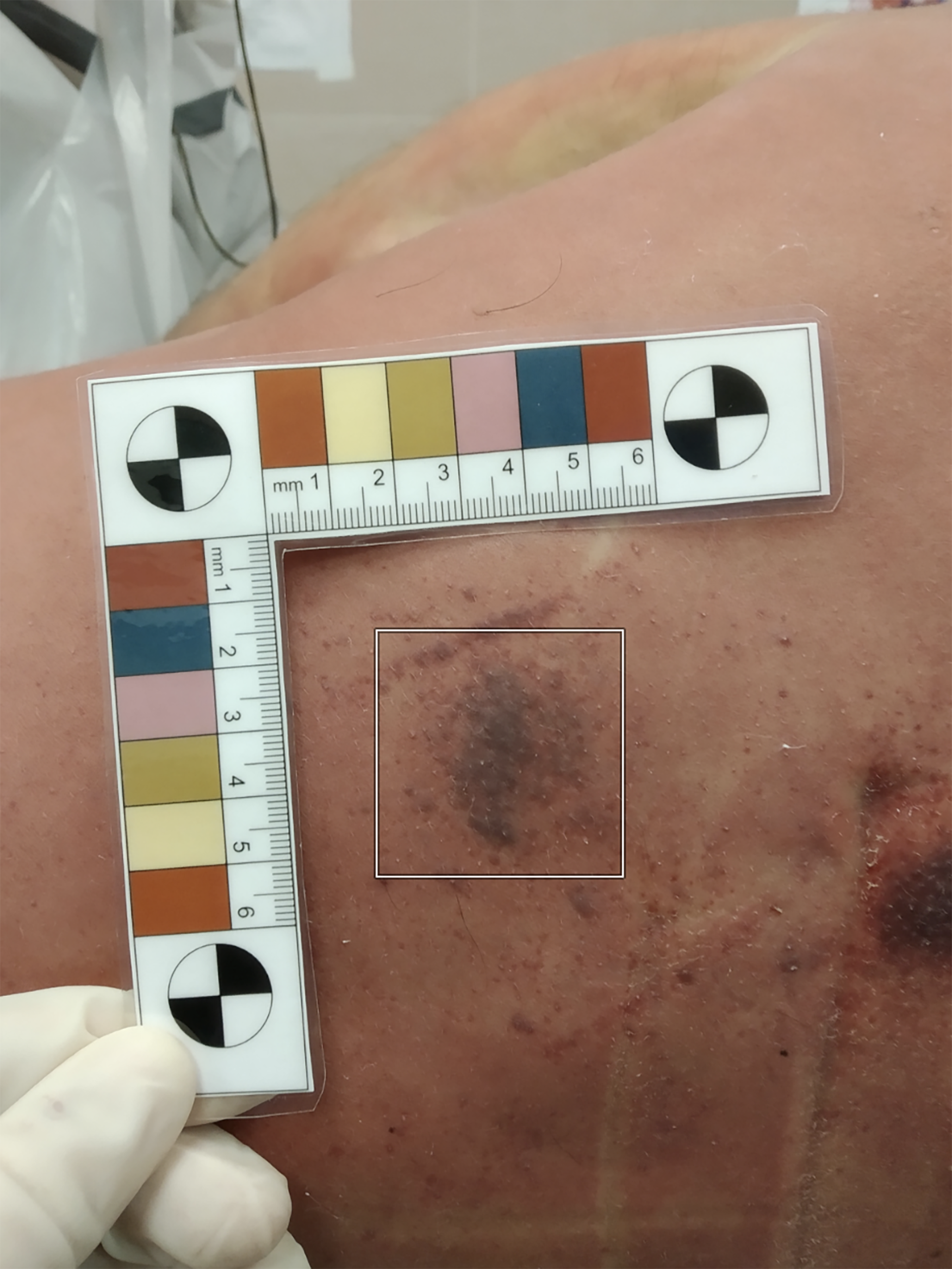


Supplemental Digital Content 1. Example of sampling of the area of interest through the GIMP eyedropper tool. An area of interest of 101 x 101-pixel is sampled on an image of about 3000 x 4000-pixel.
